# Concurrent Fetal Exposure to Multiple Environmental Chemicals along the U.S.—Mexico Border: An Exploratory Study in Brownsville, Texas

**DOI:** 10.3390/ijerph111010165

**Published:** 2014-09-29

**Authors:** Ken Sexton, Jennifer J. Salinas

**Affiliations:** University of Texas School of Public Health, Brownsville Regional Campus, 80 Fort Brown—AHC, Brownsville, TX 78520, USA; E-mail: jennifer.j.salinas@uth.tmc.edu

**Keywords:** biomarkers, chemical mixtures, cord blood, fetal exposure, health disparities, Hispanic health, organochlorine pesticides, PAHs, PCBs

## Abstract

There is mounting concern that cumulative exposure to diverse chemicals in the environment may contribute to observed adverse health outcomes in the Lower Rio Grande Valley of Texas. To investigate this situation, biomarker concentrations of organochlorine (OC) pesticides/metabolites, polychlorinated biphenyls (PCBs), and polycyclic aromatic hydrocarbons (PAHs) were measured in maternal and umbilical cord blood from pregnant Hispanic women in Brownsville, TX. Results show that both mothers and fetuses were exposed concurrently to a variety of relatively low-level, hazardous environmental chemicals. Approximately 10% of the blood specimens had comparatively high concentrations of specific OC pesticides, PCBs and PAHs. Because many pregnant women in Brownsville live in socioeconomically-disadvantaged and environmentally-challenging circumstances, there is appropriate concern that exposure to these exogenous substances, either individually or in combination, may contribute to endemic health problems in this population, including cardiovascular disease, obesity, and diabetes.  The challenge is to identify individuals at highest comparative risk and then implement effective programs to either prevent or reduce cumulative exposures that pose significant health-related threats.

## 1. Introduction

The developing fetus is known to be more susceptible to adverse toxicologic consequences of many environmental chemicals because of elevated cell proliferation rates, reduced capability to activate and/or detoxify carcinogenic chemicals, and diminished immune-response capacity, among other factors [1,2,3,4]. There is, moreover, compelling evidence that xenobiotic chemicals in maternal blood, including organochlorine (OC) pesticides [5,6,7,8,9,10], polychlorinated biphenyls (PCBs) [11,12,13,14] and polycyclic aromatic hydrocarbons (PAHs) [15,16,17,18,19,20] can cross the placenta and expose the fetus. Biological markers in umbilical cord blood are typically used to establish fetal exposure concentrations, with the vast majority of published studies reporting data for either a single chemical or chemical class [2,21,22,23]. However, there is ample evidence that the fetus is exposed routinely to a complex and constantly changing mixture of chemicals, including neurotoxicants, carcinogens, and endocrine disrupters [24,25,26,27], which may result in cumulative effects that are greater than the sum of individual toxicities [28,29,30,31,32,33,34].

One region where prenatal exposure to multiple environmental chemicals is a particular concern is along the U.S.—Mexico Border in the Lower Rio Grande Valley (LRGV) of Texas. Although actual exposure measurements are scarce, there is strong presumptive evidence that maternal/fetal exposures occur because of inadequate water supplies, marginal or nonexistent sewer systems, both indoor and outdoor air pollution, pesticide use, inappropriate waste disposal, unsafe food, and household and workplace use of hazardous chemicals [35,36,37,38,39,40]. Adding to the substantial pollution burden are a plethora of nonchemical stressors, including: rapid growth of a majority Hispanic, primarily Mexican American population; low rates of educational attainment; high rates of unemployment and poverty; a shortage of health care providers; and a general lack of awareness and knowledge about environmental health issues. Among the environmentally-related diseases and health outcomes that tend to be elevated in the LRGV are chronic liver disease, hepatitis A, tuberculosis, obesity, metabolic syndrome, diabetes, and hypertension [41,42,43,44,45,46].

In 2005–2006, an exploratory study to investigate the magnitude of concurrent fetal exposures to OC pesticides, PCBs, and PAHs (See Table 1 for common exposure routes) was undertaken in Brownsville, Texas; a city in the LRGV at the eastern end of the U.S.—Mexico border. According to the U.S. Census Bureau [47], Brownsville has a population of 172,434, with 92% of residents classified as Hispanic. In 2006, it ranked as the most impoverished city in the U.S. based on average-annual household income, and 45% of residents who were 18-years-old or younger lived in poverty—the highest proportion of any city in the country with a population over 100,000 [47]. In this article, we report on results from a biomarker study of 35 pregnant Hispanic women residing in Brownsville, including postpartum analysis of cord blood, and summarize contemporaneous biomarker measurements of 30 OC pesticides/metabolites, 22 PCB compounds or groups, and 55 PAHs.

**Table 1:** Summary of common exposure routes and biomarker interpretation for PAHs, OC pesticides, and PCBs.

Chemical Category	Likely Exposure Pathways/Sources for the General Population	Interpretation of Blood Biomarker Measurements
Polycyclic Aromatic Hydrocarbons (PAHs)	Airborne and Dietary: PAHs are produced by incomplete combustion and exposures typically occur via inhalation of contaminated air (tobacco smoke, motor vehicle exhaust) or eating smoked, grilled or broiled meats; in utero exposures and infant exposure through breast milk can occur; exposure is usually to a mixture of PAHs	Levels of PAHs have a half-life in blood of hours, so concentrations reflect recent exposures
Organochlorine (OC) Pesticides & Polychlorinated Biphenyls (PCBs)	Dietary: ingestion of fatty foods (dairy products, fish) is the primary route of exposure; minor contributors include ingestion of contaminated water or inhalation of contaminated air; in utero exposures and infant exposure through breast milk can occur; exposure is usually to a mixture of (a) OC pesticides and/or (b) PCB congeners	Levels of parent compounds or metabolites (which are lipophilic) can reflect recent or historical exposures, or both

## 2. Subjects and Methods

The study participants were recruited from pregnant women in their first or second trimester presenting at a private gynecological clinic in Brownsville. Informed verbal and written consent, either in English or Spanish, as appropriate, was obtained, and no incentives were provided to research subjects. At the time of enrollment participants completed a brief questionnaire on demographic and socioeconomic characteristics. The study was approved by the Committee for the Protection of Human Subjects at the University of Texas Health Science Center at Houston. Previous publications describe results specific for PAHs [18], OC pesticides [7], and PCBS [13].

### 2.1. Study Participants

The sociodemographic attributes of women participating in the study are summarized in Table 2. All 35 women were Hispanic, between the ages of 18 and 38 years old, and had lived in the Brownsville area for several years. Only one woman identified herself as an active smoker, but her blood PAH concentrations were unremarkable. Average height was 5.2 ft, average weight was 161 lbs, and the women, on average, had 2.6 children at home and had experienced 2.8 previous pregnancies. Sixty-three percent were born in U.S., 6% in Mexico, and 31% elsewhere or unknown. More than a quarter (28.6%) did not finish high school, and 48.6% graduated from a college or university. Sixty-three percent worked outside the home and 20% described themselves as “housewives”. Most women (71.4%) were married, while 25.7% had never been married and 2.9% were separated.

**Table 2:** Demographic and socioeconomic attributes of women participating in the study (N = 35).

VARIABLE	MEAN	STD. DEV.	RANGE
Age (years)	25.8	5.5	18–38
Height (feet)	5.2	0.21	4.8–5.6
Weight (pounds)	160.9	36.9	96–237
Previous Pregnancies	2.8	0.76	2–4
Number of Children	2.6	0.5	2–3
	**NUMBER OF WOMEN (%)**		
**Country of Birth**			
United States	22 (62.9)		
Mexico	2 (5.7)		
Other or Unknown	11 (31.4)		
**Education**			
Middle School	2 (5.7)		
Some High School	8 (22.9)		
Graduated High School/GED	7 (20.0)		
Graduated College/University	17 (48.6)		
Unknown	1 (2.9)		
**Occupation**			
Housewife	7 (20.0)		
Office/Business/Shopping Mall	14 (40.0)		
Teacher/Student/Administrator	7 (20.0)		
Outdoor Job	1 (2.9)		
Unemployed	1 (2.9)		
Other	4 (11.4)		
Unknown	1 (2.9)		
**Marital Status**			
Married	25 (71.4)		
Never Married	9 (25.7)		
Separated	1 (2.9)		

### 2.2. Specimen Collection and Handling

Sample collection occurred between October 2005 and February 2006, with venous blood samples collected during routine third-semester clinical visits and cord blood obtained at birth. Variable time elapsed between collection of maternal blood and cord blood, with 6 matched maternal-cord sample pairs obtained within 24 h of each other, 10 within 2–14 days, 16 within 15–35 days, and 3 within 43–57 days. Venipuncture was used to take maternal blood and samples were put into 10 mL, red-topped, vacutainer tubes, then labeled and refrigerated. The umbilical cord was severed at birth and approximately 10 mL of cord blood was drained into a red-topped, plain, vacutainer tube, which was capped, labeled, and refrigerated. Each unopened blood tube was sealed with Teflon tape and placed upright in an individual slot inside a pressure jar for shipping. Gel or ice packs were placed under, around, and over the jar, which was then sealed in a shipping container and sent by overnight express to the laboratory.

### 2.3. Analytical Methods

The laboratory at the Texas A&M School of Rural Public Health in College Station, TX, analyzed all samples [48]. Blood samples were analyzed (a) for PAHs using a combination of gas chromatography and mass spectrometry (GC/MS) according to modified U.S. Environmental Protection Agency (EPA) SW-846 Method 8271C [49], and (b) for OC pesticides and PCBs using gas chromatography with an electron capture detector (GC/ECD) according to modified U.S. EPA SW-846 Method 8081A [49]. All results are reported on a wet-weight basis (*i.e.*, per unit of serum). Details about sample preparation, limits of detection, procedures for dealing with not-detected and below-detection-limit values, and reporting of wet-weight *versus* lipid-adjusted concentrations have been published separately for PAHs [18], OC pesticides [7], and PCBs [13].

## 3. Results and Discussion

Maternal and cord blood specimens were analyzed for 55 PAHs, 30 OC pesticides and/or metabolites, and 22 PCBs. As shown in Table 3, 19 PAHs (34.5%) were not detected at all and 10 PAHs (18%) were not detected in ≥75% of samples. Similarly for OC pesticides and PCBs, 14 OC pesticides/metabolites (47%) and six PCBs (27%) were not detected in any specimens and 11 OC pesticides/metabolites (37%) and nine PCBs (41%) were not detected in ≥75% of samples. These compounds (39 PAHs, 25 OC pesticides/metabolites, 15 PCBs) were excluded from further statistical analysis, but are included, when appropriate, as part of “total PAH”, “total DDT”, and “total PCB” values reported in Table 4, Table 5 and Table 6.

Geometric means and standard deviations for all compounds measured in >25% of maternal and cord blood specimens (26 PAHs, 5 OC pesticides/metabolites, 7 PCBs) are summarized in Table 4, along with the ratio of cord-to-maternal blood concentrations. Observed differences between cord and maternal blood were statistically significant (*p* < 0.05) based on two-tailed t-tests for 17 PAHs and for total PAH. The nine PAHs for which concentrations were not statistically different were naphthalene, C1-naphthalenes, biphenyl, acenaphthene, C3-fluorenes, 1-methylnaphthalene, 2-methylnaphthalene, 2,6-dimethylnaphthalene, and 1,6,7-trimethylnaphthalene. Differences between cord and maternal blood were not statistically significant (*p* < 0.05) for any OC pesticides/metabolites and PCBs, including total HCH, total Chlordane, total DDT, and total PCB, based on calculated z-scores.

It is worth noting that, because 29 out of 35 cord-maternal blood pairs were collected days to weeks apart, a direct comparison of the cord-to-maternal PAH ratio implicitly assumes that PAH exposures were relatively constant over the time period that data were collected. There is no way to know for sure, but the same general pattern (*i.e.*, cord-blood greater than maternal-blood concentrations) was also observed for all six matched-sample pairs collected less than 24 h apart.

Correlations between total PAHs, total DDT, and total PCBs are presented in Table 5. Based on Pearson correlation coefficients, correlations between total PAHs and total DDT and between total PAHs and total PCBs were not statistically significant (*p* < 0.05). In contrast, correlations between total DDT and total PCBs were positive and statistically significant (*p* < 0.05) in cord blood (n = 35), maternal blood (n = 35), and all cord and maternal specimens combined (n = 70).

**Table 3:** Summary of analytes not included in the statistical analysis because they were either (a) not found at all or (b) not measurable in ≥ 75% of maternal and cord blood specimens.

No Concentration Measureable in 100% of Blood Samples		
*POLYCYCLIC AROMATIC HYDROCARBONS*		
C1-Benzothiphenes	C3-phenanthrenes/anthracenes	Benzo(k)fluoranthene
C2-Benzothiphenes	C4-phenanthrenes/anthracenes	Benzo(a)pyrene
C3-Benzothiophenes	C1-Chrysenes	Indeno(1,2,3-c,d)pyrene
Naphthobenzothipene	C2-Chrysenes	Dibenzo(a,h)anthracene
C1-Napththobenzothiophenes	C3-Chrysenes	Benzo(g,h,i)perylene
C2-Napthobenzothiophenes	C4-Chrysenes	C3-Dibenzothiphenes
C3-Napthobenzothiphenes		
*ORGANOCHLORINE PESTICIDES*		
Aldrin	2,4’-DDE	Endsulfan I
Endrin	1,2,3,4-Tetrachlorobenzene	Endosulfan II
Alpha-Chlordane	1,2,4,5-Tetrachlorobenzene	Endosulfan Sulfate
Gamma-Chlordane	Pentachloroanisole	Chlopyrifos **^a^**
Alpha-HCH	Pentachlorobenzene	
*POLYCHLORINATED BIPHENYLS*		
PCB 5/8	PCB 195/208	
PCB 18	PCB 157/173/201	
PCB 29	PCB 206	
**No Concentration Measureable in >75% to ≤99% of Blood Samples**		
*POLYCYCLIC AROMATIC HYDROCARBONS*		
Benzothiphene	C1-Fluoranthenes/pyrenes	
Acenaphthylene	C2-Fluroanthenes/pyrenes	
Carbazole	Benz(a)anthracene	
Chrysene	Benzo(e)pyrene	
Benzo(b)fluoranthene	Perylene	
*ORGANOCHLORINE PESTICIDES*		
Dieldren	Delta-HCH	
Heptachlor	Gamma-HCH	
Oxychlodane	2,4’-DDD	
Cis-Nonachlor	4,4’-DDD	
Beta-HCH	Mirex	
*POLYCHLORINATED BIPHENYLS*		
PCB 28	PCB 128	
PCB 44	PCB 170/190	
PCB 52	PCB 187	
PCB 87/115	PCB 209	
PCB 110		

**^a^** organophosphate pesticide.

**Table 4:** Summary statistics for PAH, OC Pesticide, and PCB concentrations (ng/mL) in matched pairs of cord and maternal blood specimens (N = 35).

Compound	Cord Blood GM (GSD) ng/mL	Maternal Blood GM (GSD) ng/mL	Cord/Maternal Ratio GM (GSD)
***PAHs***			
Naphthalene	1.5 (1.6)	1.5 (1.3)	1.0 (1.7)
C1-Naphthalenes	1.1 (1.6)	1.0 (1.5)	1.2 (1.6)
C2-Naphthalenes	1.5 (1.7)	0.4 (7.8)	3.8 (7.3)
C3-Naphthalenes	0.8 (4.4)	0.2 (9.5)	4.6 (16.9)
C4-Naphthalenes	0.3 (10.7)	0.1 (11.9)	2.9 (11.8)
Biphenyl	0.5 (1.7)	0.5 (1.4)	1.1 (1.9)
Acenaphthene	0.0 (3.1)	0.0 (3.5)	0.6 (4.2)
Dibenzofuran	0.4 (1.7)	0.3 (2.9)	1.6 (3.5)
Fluorene	0.3 (1.9)	0.1 (4.1)	2.9 (4.9)
C1-Fluorenes	0.7 (2.0)	0.2 (6.5)	4.3 (6.8)
C2-Fluorenes	0.9 (3.0)	0.1 (8.6)	6.0 (9.8)
C3-Fluorenes	0.1 (8.8)	0.0 (5.0)	5.0 (23.1)
Anthracene	0.1 (2.9)	0.5 (2.4)	2.5 (4.3)
Phenathrene	0.9 (1.8)	0.5 (2.4)	1.9 (2.6)
C1-P/A ^**a**^	1.0 (2.0)	0.1 (7.3)	8.0 (7.1)
C2-P/A ^**b**^	0.7 (2.8)	0.1 (7.3)	6.7 (9.6)
Dibenzothiophene	0.1 (2.5)	0.0 (3.5)	2.8 (5.2)
C1-DBT ^**c**^	0.3 (2.5)	0.1 (4.8)	6.1 (5.9)
C2-DBT ^**d**^	0.3 (3.7)	0.1 (5.0)	5.8 (6.4)
Fluoranthene	0.1 (2.1)	0.1 (3.3)	2.6 (4.0)
Pyrene	0.1 (2.6)	0.1 (3.3)	2.7 (4.9)
1-MNAP ^**e**^	0.7 (0.7)	0.7 (1.6)	1.0 (2.1)
2-MNAP ^**f**^	1.1 (0.7)	1.0 (2.0)	1.1 (3.4)
2,6-DMNAP ^**g**^	0.5 (28.7)	0.2 (1.5)	2.7 (1.8)
1,6,7-TMNAP ^**h**^	0.1 (8.4)	0.1 (1.1)	2.3 (1.1)
1-MPA ^**i**^	0.2 (10.2)	0.1 (1.3)	3.4 (1.2)
Total PAHs ^**j**^	14.1 (1.7)	7.4 (2.0)	1.9 (2.1)
***OC Pesticides***			
Heptachlor-Epoxide	0.03 (1.0)	0.03 (1.0)	1.1 (1.2)
*Trans*-Nonachlor	0.01 (1.0)	0.02 (1.0)	0.5 (4.6)
4,4’-DDE ^**k**^	0.22 (1.3)	0.82 (2.3)	0.3 (1.9)
4,4’-DDT ^**l**^	0.01 (1.0)	0.01 (1.0)	0.8 (2.3)
HCH ^**m**^	0.02 (1.0)	0.02 (1.0)	0.8 (1.8)
Total HCH ^**n**^	0.02 (1.0)	0.02 (1.0)	1.1 (5.3)
Total Chlordane ^**o**^	0.03 (1.0)	0.04 (1.0)	0.7 (3.1)
Total DDT ^**p**^	0.24 (1.3)	0.83 (2.3)	0.3 (1.9)
***PCBs***			
PCB 66	0.02 (1.0)	0.01 (1.0)	1.1 (2.5)
PCB 90/101	0.01 (1.0)	0.02 (1.0)	0.8 (2.8)
PCB 105	0.02 (1.0)	0.02 (1.0)	1.2 (2.9)
PCB 118	0.01 (1.0)	0.02 (1.0)	0.7 (3.5)
PCB 132/153/168	0.03 (1.0)	0.04 (1.0)	0.8 (5.1)
PCB 138	0.04 (1.0)	0.05 (1.1)	0.8 (2.8)
PCB 180	0.05 (1.1)	0.05 (1.1)	0.9 (3.5)
Total PCBs ^**q**^	2.67 (1.11)	2.78 (1.17)	1.0 (1.2)

GM = geometric mean; GSD = geometric standard deviation; **^a^** C1-phenanthrene/anthracene; **^b^** C2-phenanthrene/anthracenes; **^c^** C1-dibenzothiphenes; **^d^** C2-di-benzothiphenes; **^e^** 1-methylnaphthalene; **^f^** 2-methylnaphthalene; **^g^** 2,6-dimethylnaphthalene; **^h^** 1,6,7-trimethylnaphthalene; **^i^** 1-methylphenathrene; **^j^** Total PAHs = sum of all PAH concentrations >0 including those not reported in the Table; **^k^** DDE = dichlorodiphenyldichloroethylene; **^l^** DDT = dichlorodiphenyltrichloroethane; **^m^** HCH = hexachlorocyclohexane; **^n^** Total HCH = sum of all HCH isomer concentrations including those not reported in the Table; **^o^** Total Chlordane = sum of all Chlordane isomer concentrations including those not reported in the table; **^p^** Total DDT = sum of all DDT-related compounds with concentrations >0 including those not reported in the table; **^q^** Total PCB = sum of all PCB concentrations >0 (including those not reported in the table).

**Table 5:** Pearson correlation coefficients and *p*-values for associations between classes of chemicals measured in cord and maternal blood from pregnant Hispanic women in Brownsville, TX.

Association between Classes of Chemicals	Cord Blood (n = 35)	Maternal Blood (n = 35)	All Cord and Maternal Blood (n = 70)
Total PAHs and Total DDT	0.062 **^a^** (*p* = 0.722) **^b^**	−0.108 (*p* = 0.536)	−0.183 (*p* = 0.129)
Total PAHs and Total PCBs	0.324 (*p* = 0.058)	−0.071 (*p* = 0.684)	0.022 (*p* = 0.854)
Total DDT and Total PCBs	0.662 (*p* < 0.00001)	0.439 (*p* = 0.008)	0.599 (*p* < 0.00001)

**^a^** Pearson correlation coefficient; **^b^** *p*-value.

**Table 6:** Comparison of PAH, OC Pesticide, and PCB Concentrations in Blood from a National Sample of Mexican-American Adults and in Blood from Thirty-Five Hispanic Women in Brownsville, TX.

Compound(s)	CDC Exposure Report ^a^		Venuos Blood Collected from Brownsville Subjects ^b^			
	Geometric Mean	95th Percentile	Geometric Mean (GSD)	Highest Value in Maternal Blood	2nd Highest Value in Maternal Blood	3rd Highest Value in Maternal Blood
***PAHs*^c^**						
Naphthalene	NA	NA	1.5 (1.3)	3.5	1.9	1.8
C1-Fluorenes	NA	NA	0.2 (6.5)	1.5	1.0	1.0
C2-Fluorenes	NA	NA	0.1 (8.6)	2.1	2.0	1.9
C3-Fluorenes	NA	NA	0.0 (5.0)	1.8	1.5	1.4
Phenanthrene	NA	NA	0.5 (2.4)	1.4	1.3	0.8
Total PAHs	NA	NA	7.4 (2.0)	24.0	21.4	18.4
***OC PESTICIDES*^d^**						
Heptachlor-Expoxide	<LOD	0.098	0.03 (1.03)	0.19	0.17	0.14
*Trans*-Nonachlor	0.062	0.414	0.02 (1.02)	0.47	0.19	0.13
4,4’-DDE	2.69 ^**e**^	22.9 ^**e**^	0.82 (2.27)	4.45	4.40	4.34
4,4’-DDT	<LOD ^**f**^	<LOD ^**f**^	0.01 (1.01)	0.36	0.34	0.33
Total DDT	NA	NA	0.83 (2.29)	4.74	4.44	4.35
***PCBs*^g^**						
PCB 66	0.007	0.018	0.01 (1.01)	0.067	0.036	0.036
PCB 90/101	0.011 ^**h**^	0.029 ^**h**^	0.02 (1.02)	0.167	0.079	0.050
PCB 138	0.043 ^**i**^	0.239 ^**i**^	0.05 (1.05)	0.177	0.150	0.133
PCB 132/153/168	0.053 ^**j**^	0.323 ^**j**^	0.04 (1.04)	0.274	0.255	0.217
PCB 180	0.037	0.343	0.05 (1.06)	0.225	0.220	0.189
Total PCBs	NA	NA	2.78 (16.1)	3.94	3.94	3.73

**^a^*** Fourth National Report on Human Exposure to Environmental Chemicals*, Centers for Disease Control and Prevention (NRC 2009), using whole weight serum concentrations expressed in ng/g of serum or ppb; **^b^** From analysis of venus blood collected from 35 Hispanic women in Brownsville, TX, expressed as whole weight serum concentrations in ng/mL or ppb; **^c^** The CDC’s *Fourth National Report on Human Exposure to Environmental Chemicals* provides urinary metabolite concentrations instead of blood concentrations for PAHs; **^d^** From updated tables (September 2012) in the *Fourth National Report on Human Exposure to Environmental Chemicals*; data are for Mexican American adults (n = 457 – 460) collected during 2003–2004; **^e^** CDC reported concentrations for p’,p’-Dichlorodiphenyltrichloroethane; **^f^** CDC reported concentrations for o,p’-Dichlorodiphenyltrichloroethane); **^g^** From updated tables (September 2012) in the *Fourth National Report on Human Exposure to Environmental Chemicals*; data are for Mexican American adults (n = 425 to 427) collected during 2003-2004; **^h^** CDC reported concentrations for PCB 101 only; **^i^** CDC reported concentrations for PCB 138 and 158 together; **^j^** CDC reported concentrations for PCB 153 only.

Data summarized in Table 6 provide a comparison of selected analytes for the three highest (approximately the upper 10th percentile) measured maternal blood concentrations from Brownsville and a statistical sample of Mexican American adults as reported in the CDC’s (Centers for Disease Control and Prevention’s) *Fourth National Report on Human Exposure to Environmental Chemicals* [50]. Geometric mean values for OC pesticides and PCBs in the Brownsville sample were either comparable (heptachlor-expoxide, 4,4’-DDT, PCB 66, 90/101, 138, 132/153/168, 180) or lower (*trans*-nonachlor, 4,4’-DDE) than those reported by CDC. However, high-end concentrations in the Brownsville sample were higher than CDC means for heptachlor-expoxide, *trans*-nonachlor, 4,4’-DDE, 4,4’-DDT, and PCB 66, 90/101, 138, 132/153/168 and 180, and higher than CDC 95th percentile values for heptachlor-expoxide, *trans*-nonachlor, 4,4’-DDT, and PCB 66. The CDC report did not include blood concentrations of PAHs, and did not report values for total DDT or total PCBs.

### Discussion

Pregnant women and their fetuses in Brownsville, TX are exposed routinely to a diverse mixture of PAHs, OC pesticides, and PCBs. Maternal biomarker concentrations are comparable to cord blood for OC pesticides and PCBS, while cord blood levels of PAHs tend to be slightly higher. The majority of individual PAHs, OC pesticides, and PCBs were either (a) not detected at all or (b) not detected in >75% of the specimens, and mean concentrations of measurable compounds were generally relatively low. Nonetheless, approximately the upper 10th percentile of the Brownsville sample had relatively high concentrations of certain analytes, including heptachlorpexpoxide, *trans*-nonachlor, 4,4’-DDE, 4,4’-DDT, PBC 66, PCB 90/101, PCB 138, PCB 132/153/168, and PCB 180. Measured concentrations of total PAHs and total PCBs were 5- to 10-fold higher than the highest individual compound for each specimen, while total DDT levels were comparable to measured 4,4’-DDE concentrations. Total PAH values were not correlated (*p* < 0.05) with either total PCBs or total DDT, but total DDT was correlated (*p* < 0.01) with total PCBs in both maternal and cord blood.

As always, results must be interpreted in the context of study design limitations. As this was an exploratory study, we used a comparatively small (n = 35) convenience sample of pregnant Hispanic women presenting at a private OBGYN clinic, which biased the sample toward the upper socioeconomic strata (based on education and occupation). However, while the sample may be more affluent than average for Brownsville, they still live and work in a general socioeconomic environment that is disadvantaged economically, socially, and politically. It is also not known how, if at all, results were affected by the time elapsed between the collection of maternal and cord blood specimens. Furthermore, relatively low concentrations of blood biomarkers in most blood samples meant that analytical instruments were operating at or below nominal limits of detection for many compounds.

Current scientific knowledge and understanding are insufficient to determine the precise health effects that may occur from fetal exposures to this complex, real-world concoction of hazardous chemicals [1,2,3,4,21,23,30,31,32,33,34]. There are, nevertheless, two major reasons for public health concern. First, pregnant women residing in Brownsville are likely to be more vulnerable to the adverse effects of environmental exposures because the majority are Hispanic and socioeconomically disadvantaged [40,41,42,43,44,45,46,47]. Second, many of the PAHs [15,16,17,18,19,20], OC pesticides [5,6,7,8,9,10], and PCBs [11,12,13,14] observed in this study are known or suspected neurodevelopmental toxicants, human carcinogens, endocrine disrupters, and/or obesogens. The inherent biological sensitivity of the fetus, combined with the potential for synergistic reactions between various xenobiotic chemicals and nonchemical stressors, including socioeconomically-difficult and environmentally-demanding living conditions, raises serious questions about possible harmful consequences for the developing fetus and associated negative health effects in later life [1,2,3,4,21,22,23,24,28,29,30,31,32,33,34].

This study is one of the first to measure exposure biomarkers in residents of Brownsville (pregnant women in this instance). More than 90% of the population in Brownsville is Hispanic, and many individuals and families have socioeconomic attributes associated with poor health outcomes, including poverty, illiteracy, English as a second language, inadequate housing, substandard diet, lack of access to health care, and overall more stressful and less healthful lives [41,42,43,44,45,46]. Many impoverished Hispanics living along the U.S.—Mexico border tend to lack adequate sewage facilities, experience frequent flooding, live without air conditioning, not have sidewalks and street lights, and have insufficient knowledge about healthy lifestyles. Among common environmental hazards encountered by this population are (a) contaminated drinking and recreational water from agricultural runoff, municipal waste, and factory discharges, (b) adulterated soil from pesticide use, waste disposal, and illicit scrap yards and tire dumps, (c) polluted air caused by emissions from motor vehicles, industrial plants, and agricultural operations, and (d) tainted food from unhygienic growing, handling, processing, and cooking, as well as from subsistence farming and fishing [35,36,37,38,39,40]. Brownsville has a higher-than-average prevalence of several environmentally-related chronic diseases, including cardiovascular disease, tuberculosis, obesity, and diabetes [41,42,43,44,45,46], and children living along the border are hospitalized with asthma at a 36% higher rate than non-border children [51].

PCBs and OC pesticides are produced by industrial manufacturing processes, and both are toxic to humans, persist in the environment for years, bioaccumulate in the human body, and have a tendency to biomagnify in the food chain [50,52]. Consequently, their use has been banned or restricted in most industrialized countries for many years. The 2001 Stockholm Convention [52], a global treaty signed by more than 150 countries, required the elimination of production and use of PCBs and 13 OC pesticides. It also restricted the manufacture and application of DDT. But the reality is that because of continued use by some countries, along with long-range environmental transport, OC pesticides and PCBs are still routinely measured in air, water, soil, sediment, fish, birds, and mammals from all over the world [50,52]. PAHs, on the other hand, are inadvertent by-products of incomplete combustion and are virtually ubiquitous in urban and suburban settings [50,53,54]. Although PAHs are relatively short-lived in the environment, pregnant women in the U.S. are regularly exposed to PAHs in air, water, food, beverages, dust and soil [15,25,50].

Numerous human biomonitoring studies have documented OC pesticides/metabolites [5,6,7,8,9,10], PCBs [11,12,13,14], and PAHs [15,16,17,18,19,20], respectively, in both maternal and cord blood. OC pesticides/metabolites have been associated with neurodevelopmental problems [55,56,57], decreased birth size, weight and head circumference [58,59], quick weight gain and elevated BMI in infancy [5], and subsequent obesity, pre-clinical conditions, and type 2 diabetes [60,61,62,63], while PCBs are associated with neurotoxic [64,65,66], immunologic [67], reproductive [14], developmental [12,68,69,70,71], and hormonal [72,73] effects, altered glucose homeostasis, insulin resistance, metabolic syndrome, diabetes [60], and increased risk of obesity in later life [60,63,74,75]. Many PAHs are listed as “known, probable, or possible” human carcinogens [53,54], and there is evidence that PAHs affect the immune system [76], reduce fetal growth [77], and cause harmful neurodevelopmental effects [17].

While the health implications of low-level fetal exposure to a complicated combination of OC pesticides, PCBs, and PAHs is legitimate cause for concern, the aggregate effects are largely unknown [1,2,3,4,21,22,23]. Measurements of exposures have tended to focus on individual chemicals or chemical classes [21,22,23,25], and understanding of biological mechanisms underlying disease processes is limited [27,28,29,30,31,32,33]. Nevertheless, maternal exposures and related cross-placental transport remain a public health priority because the developing fetus is acutely sensitive to xenobiotic chemicals during certain time windows of vulnerability when seemingly insignificant amounts of exogenous substances can cause serious adverse effects on the fetus and/or on the course of subsequent development [1,2,3,4,21,24,27,28,31,32,33].

## 4. Conclusions

Results from this exploratory study have demonstrated that even in Brownsville’s higher socioeconomic strata (based on education and occupation), approximately 10% of pregnant Hispanic women in our sample had elevated levels of certain PAHs, OC pesticides, and PCBs in both maternal and cord blood. It is possible, if not likely, that exposures are even higher for those who are less well-off and unable to afford prenatal care at a private clinic. Given the socioeconomic privation of the Brownsville population, and the higher prevalence of many chronic diseases, follow-up research should be conducted to (a) establish the distribution of exposures, with particular emphasis on the poorest and least educated residents, (b) identify sources and pathways for those at the high-end of the exposure distribution, and (c) explore associations between exposures and prevalent chronic conditions in this population, like obesity and diabetes. A suitably large, statistically-based sample should be used and more detailed information on participants’ diets, housing characteristics, neighborhood settings, living circumstances, and activity patterns should be obtained. The overall goal should be to examine cumulative fetal exposure to multiple chemical and nonchemical stressors in the environment, with particular focus on identifying those individuals at highest comparative risk for adverse health outcomes.
